# Identification and Cross-Characterisation of Artificial Promoters and 5′ Untranslated Regions in *Vibrio natriegens*

**DOI:** 10.3389/fbioe.2022.826142

**Published:** 2022-01-27

**Authors:** Lisa Tietze, Antonia Mangold, Maria W. Hoff, Rahmi Lale

**Affiliations:** Department of Biotechnology and Food Science, Faculty of Natural Sciences, Norwegian University of Science and Technology, Trondheim, Norway

**Keywords:** *Vibrio natriegens*, *Eschecrichia coli*, artificial promoters, transcription, translation, 5ʹ untranslated region, synthetic biology

## Abstract

*Vibrio natriegens* has recently gained attention as a novel fast-growing bacterium in synthetic biology applications. Currently, a limited set of genetic elements optimised for *Escherichia coli* are used in *V. natriegens* due to the lack of DNA parts characterised in this novel host. In this study, we report the identification and cross-characterisation of artificial promoters and 5′ untranslated regions (artificial regulatory sequence, ARES) that lead to production of fluorescent proteins with a wide-range of expression levels. We identify and cross-characterise 52 constructs in *V. natriegens* and *E. coli*. Furthermore, we report the DNA sequence and motif analysis of the ARESs using various algorithms. With this study, we expand the pool of characterised genetic DNA parts that can be used for different biotechnological applications using *V. natriegens* as a host microorganism.

## 1 Introduction

*Vibrio natriegens* is an emerging non-pathogenic bacterial chassis with appealing characteristics for synthetic biology and metabolic engineering applications (Hoff et al., 2020; Blombach et al., 2021; Thoma and Blombach, 2021). The characteristics include a short doubling time of less than 10 min (Eagon, 1962); ability to produce small molecules (Hoffart et al., 2017) and recombinant proteins in high titer (Weinstock et al., 2016; Schleicher et al., 2018); tolerance to high salt concentrations and pH range (Payne et al., 1961); and ability to grow in minimal media containing different carbon sources (Hoffart et al., 2017). To date, access to reliable and characterised regulatory DNA parts is a prerequisite in synthetic biology applications. However, regulatory DNA parts are commonly characterised in a few model organisms only. To benefit from the biochemical capacity of non-model organisms, it is necessary to broaden the number of well-characterised DNA parts to include more non-model organisms (Adams, 2016). It has been proposed that *E. coli* regulatory DNA parts can be used in *V. natriegens* (Stukenberg et al., 2021). Both hosts share the same −10 and −35 consensus sequences, derived from rRNA promoters, only differing by one nucleotide in between the two motifs (Hawley and McClure, 1983; Harley and Reynolds, 1987; Aiyar et al., 2002). This similarity suggests that some genetic DNA parts, such as promoters, can be used interchangeably between the two microorganisms (Stukenberg et al., 2021). However, despite the similarity between the two hosts, the functionality of tested genetic DNA parts is not reliable. Thus far, genetic DNA parts and plasmids originating from *E. coli* have been tested in *V. natriegens* with varying success: regulatory sequences that lead to high protein production in *E. coli* do not always lead to the same results in *V. natriegens* and vice versa (Eichmann et al., 2019; Tschirhart et al., 2019; Kormanová et al., 2020). Recently, Stukenberg *et al.* characterised genetic elements to establish a ready-to-use toolkit for synthetic biology applications using *V. natriegens* (Stukenberg et al., 2021). However, to rival *E. coli* as a recombinant protein production host, the pool of available and reliable DNA regulatory sequences for usage in *V. natriegens* must be expanded further. In an earlier study, we successfully fabricated ARESs for six different Gram-negative and -positive bacteria along with Baker’s yeast using the gene expression engineering (GeneEE) method (Lale et al., 2019). The GeneEE method works through replacing native promoter and 5′ UTR with random nucleotides to drive expression of a gene of interest. In this study, we report novel *V. natriegens* ARESs leading to reliable fluorescence output. We, thus, expand the pool of characterised DNA genetic elements for this non-model microorganism. We cross-characterised 26 ARESs in *E. coli* and *V. natriegens* using super folder green (sfGFP) and red fluorescent protein (mCherry) as reporter proteins, in total 52 constructs. Additionally, we report the identification of an ARES leading to a carbon source-dependant inducible fluorescence. We conclude the study by analysing all ARESs with various promoter prediction tools to identify DNA motifs within the novel artificial sequences.

## 2 Results

### 2.1 Constructing Artificial Promoters and 5′ UTRs (ARES) Plasmid Libraries

For the identification of novel ARESs, three different plasmid DNA libraries were constructed with each library consisting of approx. 10,000 clones in *E. coli*. In each library, the native promoter and 5′ UTR sequences upstream of the reporter gene, *sfGFP*, were removed and replaced with three different sets of random nucleotides. The sets of random nucleotides were as follows: 1) 200N library, ARES comprising 200 random nucleotides; 2) 200N + SD library, ARES comprising 200 nucleotides; first 189 random nucleotides, then a fixed Shine-Dalgarno (SD) sequence, “GGAG”, followed by seven random nucleotides; 3) 50N + SD library, ARES comprising 50 nucleotides; first 39 random nucleotides, then a fixed Shine-Dalgarno (SD) sequence, “GGAG”, followed by seven random nucleotides. Concerning the SD-sequence, both hosts share the same conserved 16S rRNA 3′ anti-SD-sequence (Supplementary Figure S1). Thus, the SD-motif is expected to be functional in both hosts.

The plasmid libraries were transferred to *V. natriegens* achieving a 1.5X coverage (15,000 clones per library). Using all three libraries, we performed a small-scale preliminary library screening in *V. natriegens* with ca. 200 clones per library. We discovered that the fraction of clones emitting fluorescence differed depending on the library. About 5–10% of clones carrying library plasmids with SD-motif (200N + SD and 50N + SD) emitted fluorescence. In comparison, only 1% of clones containing the library plasmids without an SD-sequence (200N) emitted fluorescence. Therefore, for the remainder of the study, we used the two libraries that contain the SD-motif (200N + SD and 50N + SD).

### 2.2 Selection of ARESs in *V. natriegens*

**FIGURE 1:**
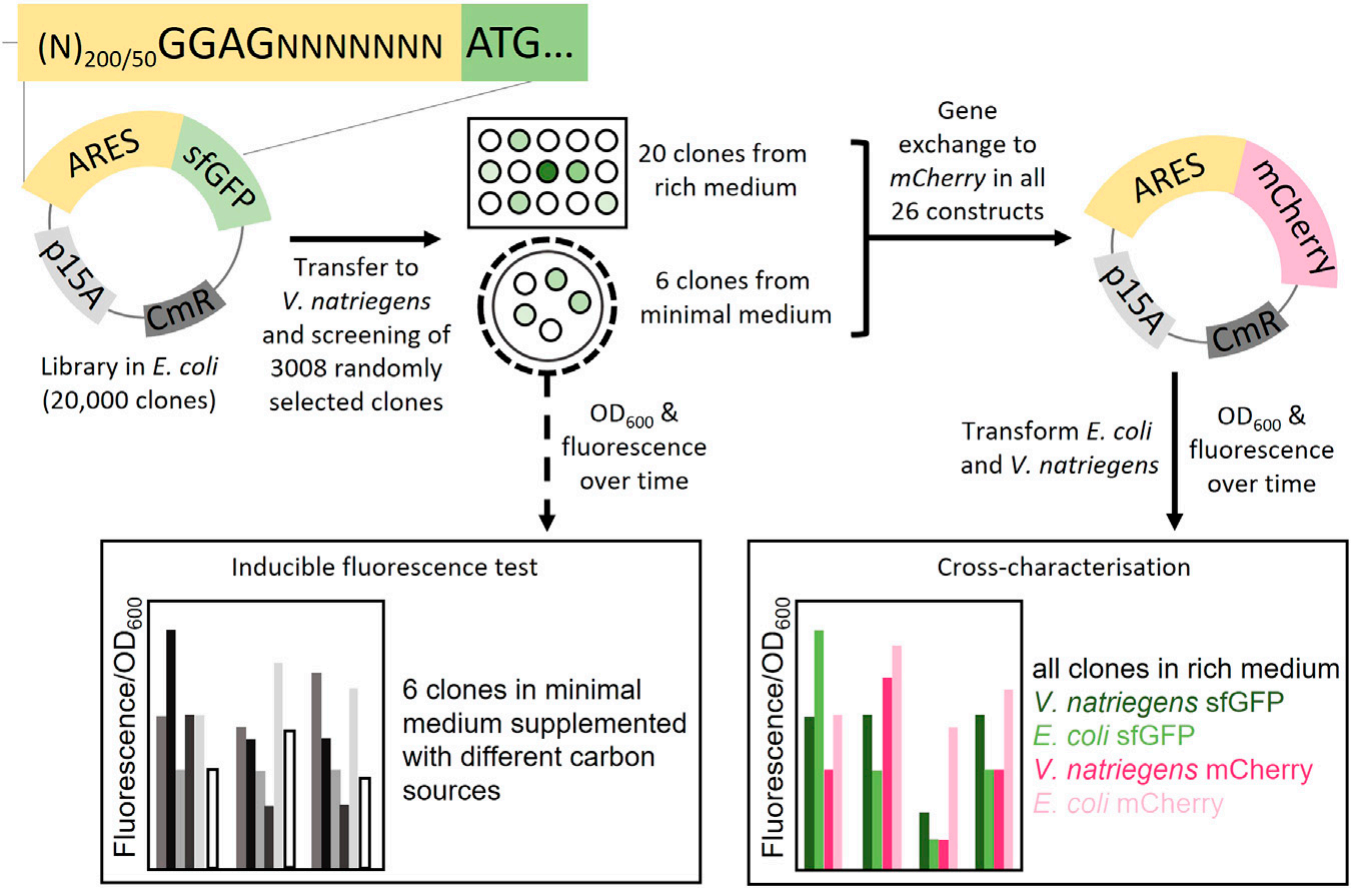
Schematic overview of the study. Plasmid libraries were constructed by cloning artificial regulatory sequences (ARES) consisting of random nucleotides upstream of the reporter gene *sfGFP*. In total three libraries were constructed, two libraries with ARES length (200 and 50 random nucleotides) with a fixed Shine-Dalgarno (SD) sequence, GGAG; and a library with only 200 nt random DNA with no SD-sequence. The plasmid libraries were screened in *V. natriegens* in two different ways: One, 3008 *V. natriegens* library clones were randomly selected from rich medium plates and cultivated in rich medium in 96-well format. The 3008 clones were screened for sfGFP fluorescence using a fluorescence plate reader. 20 clones were selected from the rich medium screening. Two, the *V. natriegens* libraries were plated out on minimal media plates. From these plates, six fluorescent colonies were visually detected and selected. The six clones from the minimal medium screening were used to conduct a inducible fluorescence test in minimal media supplemented with different carbon sources. Plasmids from all 26 constructs were extracted and *sfGFP* was exchanged with *mCherry*. Transformations were performed so that both *V. natriegens* and *E. coli* would carry all 26 constructs with each of the sets of reporter proteins (104 different clones). The clones were cross-characterised in both *E. coli* and *V. natriegens*, using fluorescence/OD_600_ as the output.

Among the 30,000 clones, from the 200N + SD and 50N + SD libraries in *V. natriegens*, we randomly selected and arrayed 3008 clones into 96-well plates and grew them in rich medium (Figure 1). Of the 3008 clones, 2500 contained 200N + SD library plasmids while the remaining 508 contained 50N + SD library plasmids. We screened the 3008 clones using sfGFP as the reporter protein. The randomly selected clones exhibited a wide range of fluorescence intensities (FIs, fluorescence/OD_600_) (Supplementary Figures S2A,B). The functional clones could also be visualised using a fluorescent microscope (Supplementary Figure S3). From the randomly selected clones, 6.5% exhibited at least 50% FI of the positive control. Among the fluorescent clones, we selected 20 unique clones with varying sfGFP FI. Using Sanger sequencing, we sequenced the part of the plasmid containing the ARES. Based on the sequencing results, we determined ARES length, which was 50–307 nt (Supplementary Table S1). The SD-motif was approximately in the expected location within the ARESs. The deviation of sequence length from the expected 50 or 200 nt is caused by the random DNA synthesis process (see Materials and Methods for more details). We noticed that eleven ARESs had in-frame alternative ATGs upstream of the planned start codon when we examined the DNA sequences (Supplementary Table S1). It is possible that the upstream ATG may serve as an alternative translation start site (TnSS) for those constructs. In addition, nine of these eleven ARESs had an SD-like motif upstream of the alternate in-frame ATG (Supplementary Table S1). This upstream SD-like motif could serve as an additional ribosome binding site (RBS). The presence of an alternate SD-like motif raises the possibility that the alternative in-frame ATG is a functioning alternative TnSS. To determine how likely it is that these alternative TnSSs are functional, we used the RBS Calculator (Salis et al., 2009) to predict translation rates from all possible TnSSs. Translation rates of alternative TnSSs were predicted to be lower than translation rates of the intended TnSS for all constructs except for sfGFP ARES 20, which was predicted to have a similar value for both TnSS (Supplementary Table S2). This implies that for at least ten of the eleven constructs with an alternate in-frame upstream ATG, the gene start is the real TnSS.

### 2.3 Characterisation of ARES in *E. coli* and *V. natriegens*

**FIGURE 2:**
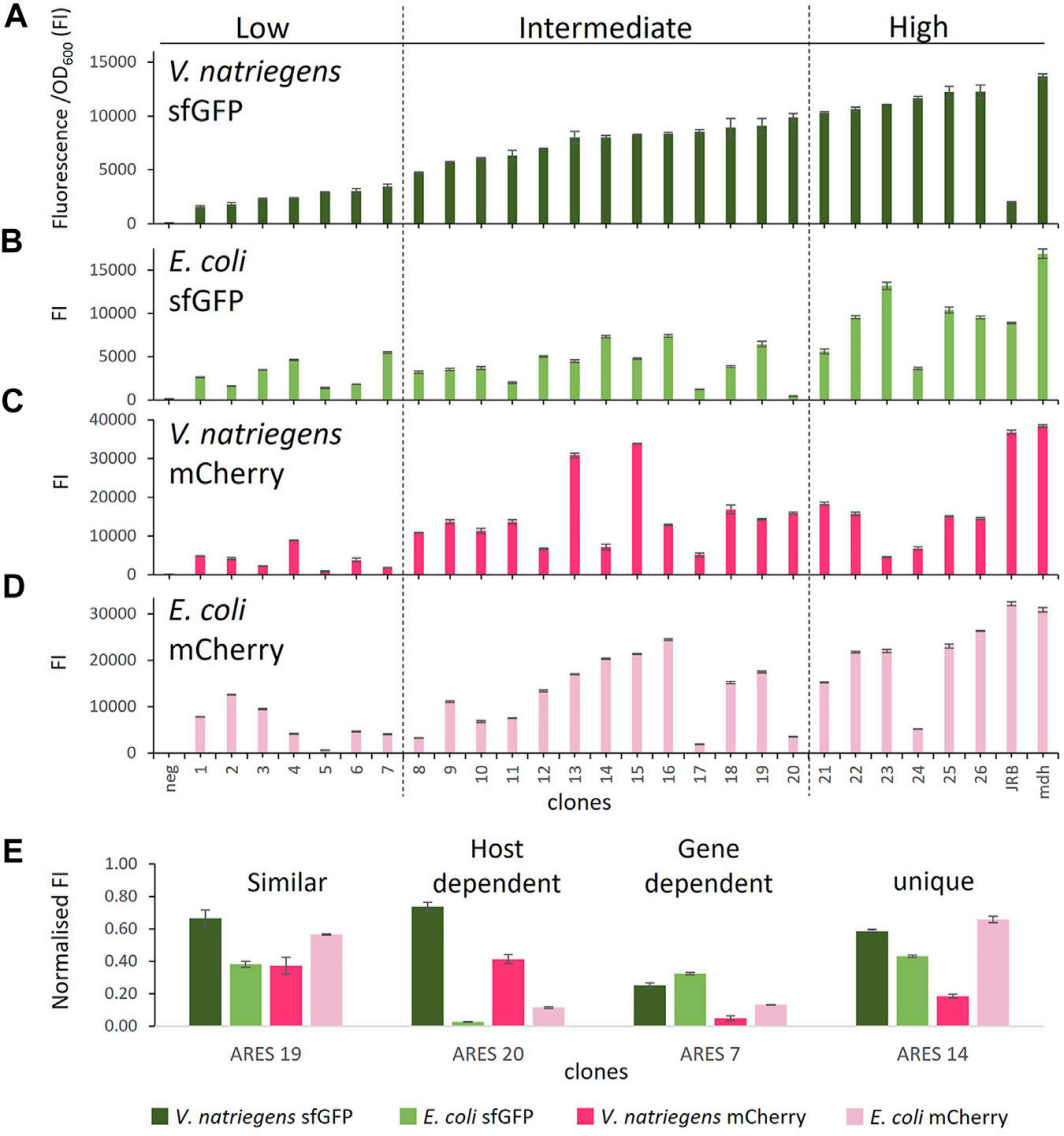
Comparison of FI in the different host-reporter combinations grown in rich medium: **(A)** *V. natriegens* clones expressing sfGFP, **(B)** *E. coli* clones expressing sfGFP, **(C)** *V. natriegens* clones expressing mCherry, **(D)** *E. coli* clones expressing mCherry, **(E)** clones representing different observed expression patterns. FI* stands for fluorescence intensity = fluorescence/OD_600_. The clones were arranged by sfGFP FI in *V. natriegens* and divided into three categories based on low, intermediate, and high FI compared to the positive control. Error bars indicate the standard deviation of three biological replicates. The outline of the figure was inspired by Wu *et al.* Wu et al. (2020).

To assess the coding sequence dependency of the identified 20 ARESs, we substituted *sfGFP* with *mCherry*. All 40 constructs were cross-characterised in *E. coli* and *V. natriegens* using both sfGFP and mCherry as reporters (Figure 1). Two positive controls were established: 1) pACYC-mdh-reporter, which comprises the *mdh* promoter, and 5′ UTR (Jakobsen et al., 2006; Irla et al., 2016) upstream of the reporter gene; and 2) pACYC-JRB-reporter, which comprises Anderson promoter J23101^1^, ribozyme RiboJ (Lou et al., 2012), and a ribosome binding site B0046^2^ upstream of the reporter gene (Shao et al., 2021). The positive controls led to similar FIs for mCherry. However, for sfGFP, pACYC-JRB-sfGFP led to lower FI in both hosts (Figures 2A,B). Thus, we used pACYC-mdh-reporter FI as the primary reference for FI measurements. The negative control constructs contained the reporter gene without an upstream 5′ regulatory sequence (no promoter and 5′ UTR), leading to negligible fluorescence emission (Figures 2A–D).

We initially sorted and named *V. natriegens* clones by their sfGFP FI levels and divided them into groups of low, intermediate and high FI (Figure 2A). We next compared the FIs of the various host-reporter combinations grown in rich medium. It is important to note that *E. coli* and *V. natriegens* grow to different OD_600_, and sfGFP and mCherry absolute fluorescence strengths vary. We, therefore, normalised FIs in relation to the appropriate host-reporter positive control, which is set to 1. Comparing normalised FIs of the different host-reporter combinations led to four distinct groups: 1) similar FI in both host-reporter combinations, 2) gene-dependent FI, 3) host-dependent FI, 4) unique FI (examples of each group in Figure 2E). We found eleven constructs (4, 6, 9, 10, 16, 18, 19, 21, 22, 25, 26) that exhibited similar normalised FI in both hosts and for both reporters. Of these, the clone carrying ARES 22 grew poorly when expressing mCherry in *E. coli*. The experiment was repeated, but the findings did not improve (data not shown). Poor growth could be caused by a heavy metabolic burden imposed by high reporter protein production. We furthermore identified three ARESs (5, 7, 15) leading to gene-dependent expression, indicated by higher FI of one reporter in both hosts. The individual clones carrying the constructs ARES 5 and 7 exhibited higher normalised sfGFP FI in both hosts, and clone carrying ARES 15 construct exhibited higher normalised mCherry FI. Moreover, five ARESs (8, 11, 13, 17, 20) led to host-dependent expression pattern, meaning both reporters exhibited higher normalised FI in one of the hosts. All five host-dependent ARESs led to higher normalised FI in *V. natriegens*. Notably, *V. natriegens* carrying pACYC-reporter ARES 20 emitted 4,5-times (mCherry) and 23-times (sfGFP) higher normalised FI than *E. coli* carrying the same construct. Lastly, seven clones (1, 2, 3, 12, 14, 23, 24) displayed unique normalised FIs in the different host-reporter combinations.

### 2.4 Testing ARESs for Inducible sfGFP Fluorescence

After observing that we could identify ARESs that lead to constitutive expression, we sought to assess if ARESs can lead to inducible sfGFP expression in *V. natriegens* in the presence of different carbon sources. For this assessment, *V. natriegens* carrying 200N + SD and 50N + SD libraries were plated on minimal media plates containing different carbon sources (glucose, fructose, sucrose, succinic acid, maltose, glycerol, arabinose, glucosamine hydrochloride). The plates were assessed every day for fluorescence development of colonies. Colonies that showed fluorescence after four days were selected. Plasmids were extracted and the ARESs were sequenced. From this minimal medium screening, we obtained six unique constructs (pACYC-sfGFP ARES 2-7), which were characterised in minimal medium as described below, and cross-characterised as described in the previous section.

**FIGURE 3:**
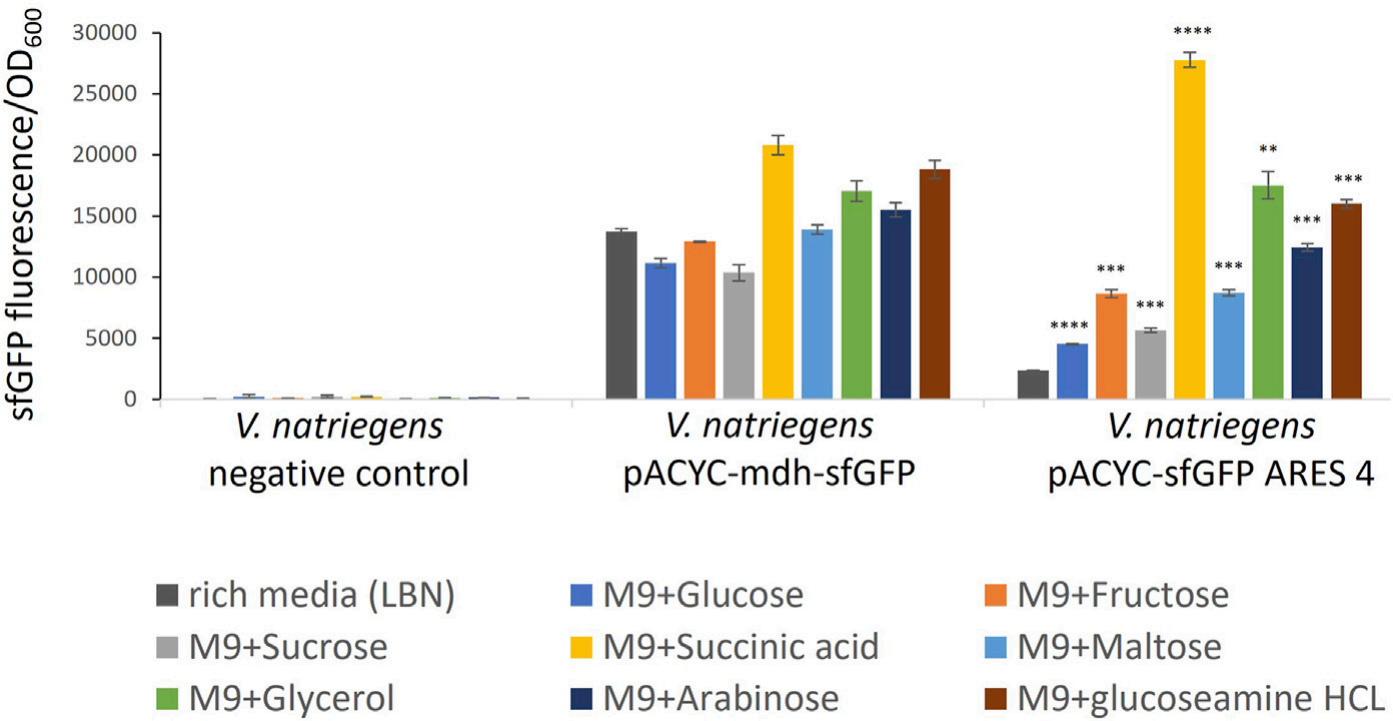
Comparison of sfGFP FI of *V. natriegens* cultivated in M9N supplemented with different carbon sources as indicated by colour. The negative control exhibits negligible fluorescence intensity. FI of the positive control and the clone carrying pACYC-sfGFP ARES 4 changes in a medium-dependent fashion. Error bars indicate the standard deviations calculated from three biological replicates. Asterisks indicate results of one-tailed T-tests, comparing FI measured in rich medium to minimal media of *V. natriegens* carrying pACYC-sfGFP ARES 4: *p-value < 0.05; **p-value < 0.01; ***p-value < 0.001; ****p-value < 0.000 1.

First, cultivation with two different minimal media (modified CGXII and M9N) was tested to identify which minimal media was most suitable for the experiment. Cultivation in M9N minimal medium resulted in better reproducibility and, thus, was used for the experiment. We cultivated *V. natriegens* pACYC-sfGFP ARES clones 2-7, the negative control and positive control carrying pACYC-mdh-sfGFP in minimal medium. We used eight different carbon sources to supplement M9N with: glucose, fructose, sucrose, succinic acid, maltose, glycerol, arabinose, and glucosamine hydrochloride. Growth and sfGFP fluorescence was followed over time. The negative control emitted negligible amounts of fluorescence (Figure 3). We compared FI at the end of exponential growth phase of clones cultivated in minimal medium to clones cultivated in rich medium (Figure 3, Supplementary Figure S4).

Five of the six tested clones exhibited the same or lower FI when cultivated in minimal medium compared to rich medium (Supplementary Figure S4). However, *V. natriegens* carrying pACYC-sfGFP ARES 4 exhibited up to 11.7-fold (*p* < 0.000 1) increased FIs (Figure 3). In M9N + succinic acid, FI was higher than FI of the positive control in either rich medium or M9N + succinic acid. For comparison, in rich medium, *V. natriegens* carrying pACYC-sfGFP ARES 4 exhibited 5.9-fold lower FI than the positive control (*p* < 0.001), while exhibiting 1.33- fold higher FI than the positive control in M9N + succininc acid (*p* < 0.001). This demonstrates that ARESs can be fabricated which exhibit inducible fluorescence in the presence of certain carbon sources.

### 2.5 Comparing Sigma Factor Protein Sequences of *E. coli* and *V. Natriegens*

An ARES contains both promoter and 5′ UTR sequences. To identify the theoretical location of the transcription start site (TSS), hence the promoter and 5′ UTR sequences, we sought to analyse all identified ARESs with promoter prediction tools. However, currently there are no promoter prediction tools that can specifically identify *V. natriegens* promoters. Given that all promoter prediction tools rely on the known motifs for *E. coli* sigma factors, we wanted to assess how (theoretically) reliable these tools could be for also predicting *V. natriegens* promoters. Therefore, we compared the sigma factors of *E. coli* and *V. natriegens*. Sigma factors associate with DNA polymerase to build the holoenzyme. The sigma factor that associates with the holoenzyme determines which promoter motif the holoenzyme binds to. If the sigma factors of two organisms are similar, the sigma factors should bind to comparable promoter motifs. Thus, if the sigma factors of *E. coli* and *V. natriegens* are similar, promoter prediction tools designed for *E. coli* may be utilised to predict promoters of *V. natriegens*.

We aligned sigma factor protein sequences of *V. natriegens* (accession ID: CP016345 and NZ_CP016346.1) to protein sequences of the corresponding sigma factors of *E. coli* (accession ID: U00096.3). The genome of *V. natriegens* contains genes encoding for six of the seven sigma factors that are present in *E. coli*. Five of the sigma factors belong to the σ^70^-family which recognise and bind to specific sequences in the –10 and –35 region of promoters (Wigneshweraraj et al., 2008) (alternative sigma factor names in brackets): sigma factors RpoE (σ^24^), RpoF (or FliA) (σ^28^), RpoH (σ^32^), RpoS (σ^38^), RpoD (σ^70^). Additionally, genomes of *V. natriegens* and *E. coli* contain genes encoding for the sigma factor RpoN (σ^54^), whose protein structure is distinct from the σ^70^-family and recognises and binds to the –12 and –24 region of promoters (Wigneshweraraj et al., 2008). We aligned protein sequences of the sigma factor pairs globally (complete protein sequence) and locally (promoter recognition and binding regions only) using EMBOSS Stretcher for pairwise sequence alignments^3^. EMBOSS Stretcher provides scores for amino acid (AA) identity and similarity for the alignments. Identical AAs are the exact same AA in the same relative position in the alignment, while similar AAs have similar biochemical properties, which can serve the same biological function. The global sequence alignment of the sigma factors displayed identity and similarity scores as low as 44 and 59% for RpoF (Supplementary Table S3). The local alignment of region 2.4 and 4.2, which are the σ^70^-family subunits of that recognise and bind to promoters (Lonetto et al., 1992; Barchinger et al., 2012; Lim et al., 2019) show higher identity and similarity scores than the rest of the protein. RpoF scored the lowest with 75% identity and 90% similarity (region 2.4), and 69.7% identity and 78.8% similarity (region 4.2). Global alignment of the distinct RpoN protein sequence resulted in 62% identity and 76.9% similarity, while the local sequence alignment of RpoN promoter binding regions (Doucleff et al., 2007) scored 75% identity or higher. Based on the estimated relatively high similarities, we decided to employ the *E. coli* promoter prediction methods to predict *V. natriegens* promoters.

### 2.6 ARES Analysis

To analyse all identified ARESs, we first searched for existing similar sequences using the NCBI Basic Local Alignment Search Tool (BLAST)^4^. For all sequences no significant similarity was found. For promoter predictions, we used the online software BPROM (Solovyev and Salomov, 2011)^5^, promoter calculator from the Salis lab (Fleur et al., 2021) and CNNPromoter_b (Umarov and Solovyev, 2017)^6^. The tools indicate the −10 region, −35 region and TSS (BPROM and CNNPromoter_b) or calculate transcription rate scores for each nucleotide (promoter calculator). The output of the three tools was very similar. The predicted location of the TSS was either at the same position or within three nucleotides for the majority of non-outlier data (Supplementary Table S4). The results from CNNPromoter_b were used for downstream analysis. The choice of CNNPromoter_b was based on a recent publication by Cassiano and Silvia-Rocha who compared the performance of several promoter prediction tools such as CNNProm, BPROM and BacPP (Cassiano and Silva-Rocha, 2020). CNNPromoter_b-predicted promoter sequences were fed into iPromoter-2L, which is an online tool that an be used to predict which sigma factor binds to a promoter. Fourteen of the artificial promoter sequences were predicted to be recognised by either RpoD or RpoE (Supplementary Table S1). Twelve promoters were not recognised as a promoter sequence by iPromoter-2L and had no sigma factor assigned to them. Among the twelve unrecognised promoter sequences were the three best expressing constructs in the host-reporter combination *V. natriegens*-sfGFP.

**FIGURE 4:**
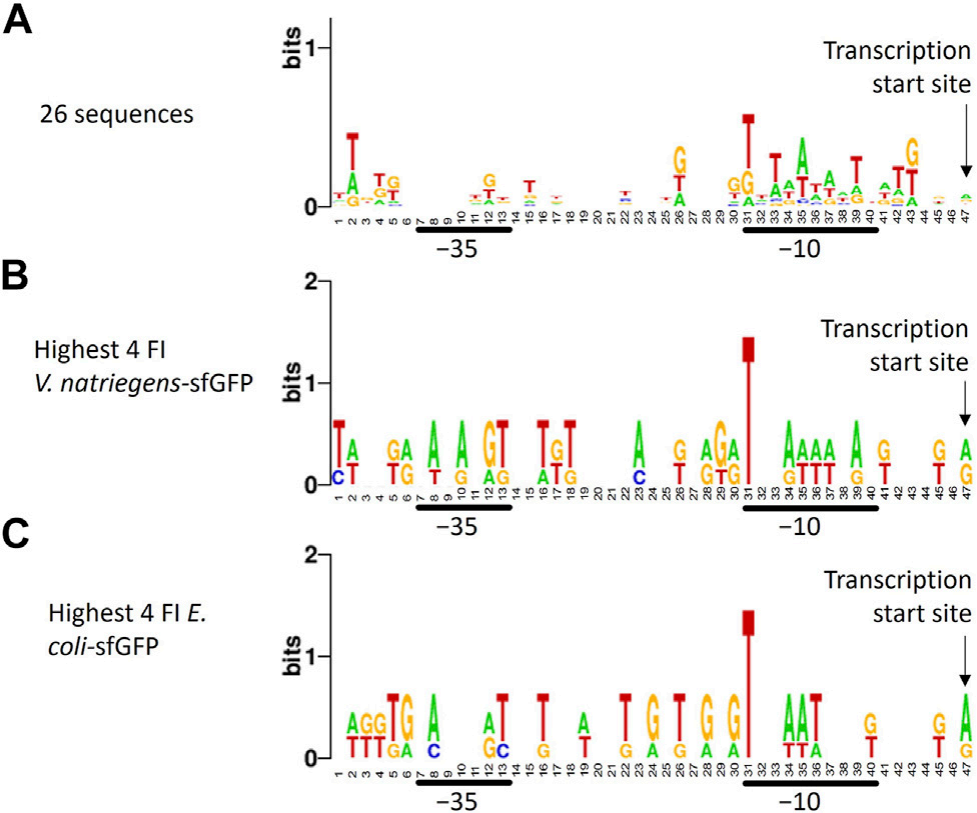
Sequence logo created from trimmed aligned predicted promoter sequences. The sequence logo represents nucleotide biases in specific positions by size. The taller the nucleotide, the more frequently the nucleotide appeared in that positions of the input sequences. Different alignments were made: **(A)** all 26 sequences, **(B**) promoter sequences of the four *V. natriegens* clones with highest sfGFP FI (clones 23, 24, 25, 26), **(C)** promoter sequences of the four *E. coli* clones with highest sfGFP FI (clones 22, 23, 25, 26). The transcription start site, -10 and -35 regions are indicated. A minute nucleotide-position bias can be seen in the -10 region when all sequences are aligned **(A)**. Aligning sequences of clones with the highest sfGFP FI reveals a bias towards T in position 31, which is the -10 box start nucleotide.

To identify recurring promoter motifs, we aligned ARESs at their theoretical TSS and created a sequence logo using Weblogo^7^. When aligning the sequences, a minute consensus sequence emerged in the −10 region: T_TAATA_T (Figure 4A). This minute consensus sequence closely resembles the *E. coli* −10 motif TATAAT, also called Pribnow box. We furthermore aligned promoter sequences of different subsets of clones: the four clones exhibiting highest and lowest FI of each host-reporter combination. When aligning promoter sequences of clones with highest sfGFP FI in *E. coli* and *V. natriegens*, we found T-bias in position 31, which is the start of the −10 motif in the sequence logo (Figures 4B,C). We did not observe position 31 T-bias for the lowest sfGFP intensity clones (Supplementary Figures S5E,F). Aligning highest and lowest mCherry producers also revealed no nucleotide bias (Supplementary Figures 5A–D). No further reoccurring motifs could be found outside of the −10 region.

We furthermore investigated whether transcription rates predicted by the promoter calculator (Fleur et al., 2021) or translation rates predicted by the RBS calculator (Salis et al., 2009) correlated with the measured FI values. If the data correlates well, it might be possible to predict FI based solely on the DNA sequence. We plotted the data and used Spearman’s rank correlation coefficients (*r*_*s*_) to determine how well the predicted and measured values correlate (Supplementary Figure S6). The estimated transcription rates correlated poorly with the experimental data (*r*_*s*_ < 0.32; Supplementary Figure S6A) and therefore could not be used to identify FIs. The predicted translation rates correlated to some degree with the experimental data (*r*_*s*_ = 0.3–0.62; Supplementary Figures S6B,C) and could potentially be used to indicate FIs, but not reliably.

## 3 Discussion

In this study, we identified and cross-characterised ARESs leading to expression of fluorescent reporter proteins sfGFP and mCherry in the novel chassis of *V. natriegens* and model organism *E. coli*. None of the ARESs could be matched to existing entries in NCBI, indicating that the sequences are unique. We demonstrate that the ARESs are functional in *V. natriegens*, and thereby, widen the spectrum of known and characterised regulatory sequences for use in *V. natriegens*. Despite a small sample size of 3008 clones, we were able to find clones emitting a variety of FI and even a clone with carbon-source-dependent inducible sfGFP phenotype.

Our results suggest that protein production in *V. natriegens* may be SD-motif-dependent. *V. natriegens* carrying 200N + SD library plasmids displayed a 5–10-fold higher positive hit rate than *V. natriegens* carrying 200N library plasmids without an SD-motif. This contrasts our findings in *E. coli*, where we found similar positive hit-rates for libraries with and without an SD-motif (Lale et al., 2019). *E. coli* harbours SD-independent expression mechanisms (Fargo et al., 1998; Saito et al., 2020) and for some organisms the SD-motif is redundant for protein synthesis (Chang et al., 2006; Zheng et al., 2011; Nakagawa et al., 2017). Thus, we propose that translational control is more stringent in *V. natriegens* than *E. coli*. We conclude that it is possible to randomise the regulatory region and drive gene expression in *V. natriegens*. However, targeting core regulatory region elements strongly affects expression, which has also been found by Wu *et al.* (Wu et al., 2020). Wu *et al.* reported similar work by randomising either the −10 motif and −35 motif, the spacer in between the two motifs, or parts of the RBS. The group found that expression was affected negatively when conserved motifs such as the −10 motif, −35 motif and RBS were randomised. Expression was improved when the spacer region was targeted. In comparison, we randomised a much larger stretch of sequence including the −10 motif and −35 region. We were nevertheless able to find constructs with almost the same FI as the positive control pACYC-mdh-reporter and higher FI than the positive control pACYC-JRB-sfGFP. This illustrates that when conserved motifs are unknown, the GeneEE technique can be particularly useful in constructing functioning ARESs.

It is critical for synthetic biology applications to have access to regulatory sequences that drive expression with predictable results and can be utilized as references to compare expression levels to. When establishing the positive controls, we found that pACYC-JRB-reporter constructs led to unpredictable behaviour. Clones carrying pACYC-JRB-reporter exhibited low level FI when expressing sfGFP and high level FI when expressing mCherry (Figure 2). The promoter and 5′ UTR of the JRB constructs comprised Anderson promoter J23101, RiboJ and RBS B0064. Using J23101 on a p15A-replicon plasmid has been proposed as a promoter reference by Kelly *et al.* (Kelly et al., 2009), and has recently been used by Shao *et al.* to express YFP in *V. natriegens* using the same regulatory region as was used in this study (Shao et al., 2021). Moreover, Stukenberg *et al.* recently described a high correlation of Anderson promoter strength between *V. natriegens* and *E. coli* (Stukenberg et al., 2021). It is unclear why sfGFP was expressed poorly with the JRB regulatory region and in contrast well with the *Bacillus methanolicus* derived *mdh* regulatory region, but it shows that, 1) there is a need for regulatory sequences adapted for the use in *V. natriegens* and, 2) regulatory sequences optimised for *E. coli*, though generally functional in *V. natriegens*, may behave unpredictably and thus need to be characterised to be useful for synthetic biology applications with *V. natriegens*.

When cross-characterising the 52 constructs, we searched for ARESs leading to reliable expression in all host-reporter combinations since predictability is an important characteristic for reusable regulatory sequences. We are confident that using sfGFP and mCherry as reporters suffices to test if an ARES is gene- and/or host-dependent. The composition of AAs and mRNA may influence translation in a variety of ways. For example, the composition of AAs and codons affects translation speed *via* interactions with the ribosome (Verma et al., 2019). Furthermore, mRNA stability affects mRNA half-life times and thus how often one strand of transcript will be translated (Kudla et al., 2009). Secondary mRNA structures may limit access of the ribosome to transcripts (Kudla et al., 2009). mRNA GC-content influences translation due to the so called ramp effect (Tuller et al., 2010). Hence, using two proteins, which differ in AA composition and mRNA level, should suffice to test reliability of the ARESs. Due to experimental selection bias, we anticipated that the majority of ARESs would be gene-dependent. Clones were chosen based on their ability to produce sfGFP. In the diverse host-reporter combinations, however, only four of the 26 ARESs resulted in gene-dependent expression and 11 ARESs resulted in similar normalised FIs. This implies that ARESs generated using the GeneEE technique have the potential to work reliably in a variety of genomic contexts.

We identified five ARESs leading to host-dependent expression. In all five cases, normalised FI in *V. natriegens* was higher than in *E. coli*. Chassis-dependent expression can be valuable when *E. coli* is used as a cloning host of toxic genes, but is not the intended production host. Cloning genes which are toxic to *E. coli* affects cell viability and disrupts the cloning process (Saïda et al., 2006). In an earlier study we reported that *E. coli* readily utilises up to 40% of random nucleotide sequences to drive gene expression (Lale et al., 2019). This can be problematic, when *E. coli* is not the intended production host. Therefore, it is useful to have access to target host specific promoters and 5′ UTRs which do not lead to expression in *E. coli*. Even though the transcription machinery is highly conserved among *V. natriegens* and *E. coli* (Stukenberg et al., 2021) we found that 19% of characterised ARESs displayed host-dependent expression with higher expression in the target host than *E. coli*. As a result, we believe that such host-specific ARESs can be developed for other organisms as well.

We furthermore investigated if we could fabricate ARESs that lead to inducible fluorescence. We found that ARES 4 led to a significant increase in sfGFP FI when cultivated in minimal media supplemented with different carbon sources compared to rich medium. The increase in FI indicates that ARES 4 is responsive to the presence of the carbon source or its derivatives in the medium. However, further research is needed to investigate what causes the increase in FI. We previously have shown that it is possible to create inducible ARESs for *E. coli* (Lale et al., 2019) and demonstrate here that it is also possible for *V. natriegens*.

Beside experimental cross-characterisation of the constructs, we analysed the ARESs. To date, promoter characterisation tools tailored to *V. natriegens* are unavailable. However, we found that the promoter DNA recognition and binding sites of the sigma factors of the two hosts are highly similar on protein sequence level, suggesting that sigma factors of both hosts recognise and bind to similar promoter motifs. The hosts also share the −10 and −35 consensus sequence of their respective rRNA promoters with a slight difference in spacing: the distance between −10 and −35 motifs is 16 nucleotides in *V. natriegens* and 17 nucleotides in *E. coli* (Harley and Reynolds, 1987; Aiyar et al., 2002; Hawley and McClure, 1983). The results from the sigma factor protein sequence alignments suggested that we could use promoter characterisation tools tailored to *E. coli* to analyse the ARESs and determine the TSS. When aligning promoter sequences, we found that the −10 region was A/T enriched as is common for native promoters of *E. coli* (Pribnow, 1975). The highest sfGFP producers in *V. natriegens* and *E. coli* showed a T-bias for the start of the −10 box (position 31, Figures 3B,C), not present in the lowest producer, indicating that T in this position may be important for strong promoter activity. Eleven sequences contained upstream in-frame ATGs, which could function as alternative start codons. It is unclear how many of these alternative start codons are productive start codons. It is a drawback of the GeneEE method that such upstream in-frame ATGs can exist. This is something to be aware of when using the method to produce proteins, since it could add unwanted leader peptides to the protein of interest. Results from iPromoter-2L indicated that fourteen of the promoters are targeted by either RpoD or RpoE. We expected high occurrence of RpoD motifs since RpoD is the housekeeping sigma factor in *E. coli* and *V. natriegens*. We did not expect occurrence of RpoE motifs, since RpoE is associated with heat-shock response (Rouvière et al., 1995). *V. natriegens* was originally isolated from the salt marsh muds of Sapelo Island (Payne, 1958). The Sapelo Island is subjected to temperatures ranging from 0°C to 35°C. The monthly average lies between 15 and 28°C^8^. Thus, growing an organism from the Sapelo Island at 37°C in laboratory conditions may cause a heat shock response even though 37°C is the optimal cultivation condition (Lee et al., 2019). Still, heat shock response could explain unexpected occurrence of promoters that are recognised by heat-shock response associated sigma factor RpoE. Twelve of the promoters had no sigma factor assigned to them, indicating that iPromoter-2L cannot identify where a sigma factor should bind. Thus, artificial sequences could be a useful source of sequences to feed into predictive models such as iPromoter-2L to increase their predictive strength for a wider sequence spectrum.

Since DNA recognition and binding sites in *E. coli* and *V. natriegens* are highly similar and the anti-SD-sequence is the same, we expected regulatory sequences to drive expression to a similar degree in *E. coli* and *V. natriegens*. In fact, all ARESs led to protein production in all host-reporter combinations. On average, normalised FI per ARES varied by less than 2-fold from each other in the different gene-host combinations. However, when considering the individual constructs, we can see that the FI varies by as much as 23-fold between both hosts carrying the same plasmid. We were unable to use predicted transcription or translation rates as an indicator for FI. It is commonly accepted that transcript abundance is a better indicator for protein abundance than translation rates are (Liu et al., 2016). However, transcription rates did not correlate well with FIs. This suggests that experimental data produced through the use of ARESs can be valuable for training and improving prediction tools such as the promoter calculator. The same is true for the RBS Calculator. Predicted translation rates correlated better with measured FIs than transcription rates did, but the correlation was still only mediocre. Moreover, correlation values varied between the different host-reporter pairs, indicating that predicting FIs from translation rates is unreliable. Furthermore, were predicted translation rates exactly the same whether selecting *E. coli* or *V. natriegens* as a host organism, suggesting, that the same algorithm is used to predict translation rates for the two organisms. Indeed, translation mechanisms are similar in both hosts. Both organisms share the anti-SD-sequence 5′ -ACCUCCUUA-3′ (Supplementary Figure S1). However, it has been found that mRNA to protein ratios are higher in *V. natriegens* than in *E. coli* suggesting that *V. natriegens* produces less protein per mRNA than *E. coli* does (Aiyar et al., 2002). At the same time, *V. natriegens* needs to synthesise proteins at a high rate, due to its short doubling time. Aiyar *et al.* suggested, that increased ribosome abundance compensates for the low protein per mRNA production (Aiyar et al., 2002). The group estimated that if *E. coli* were growing at the same rate as *V. natriegens* (4 doublings per hour), it would contain 90,000 ribosomes/cell, while *V. natriegens* was estimated to contain 115,000 ribosomes/cell. The difference in ribosome abundance and mRNA to protein ratios may factor into why the ARESs did not drive expression to a similar degree in *E. coli* and *V. natriegens*. Similarly, this fundamental biological difference between the two organisms should factor into translation rate predictions made by the RBS calculator, which currently gives the same output for *E. coli* and *V. natriegens*.

Our experiments show that introducing the same plasmid into *E. coli* and *V. natriegens* may lead to different FI produced by the two hosts. Further experiments, such as qPCR and experimental transcription start site determination could help to clarify to what extent the two hosts differ in the transcription and translation processes causing the difference in expression levels. However, the exact processes causing observed behaviour of the clones does not need to be known. The GeneEE method can be used to create functional ARESs without prior knowledge of conserved motifs, and transcriptional and translational processes, as demonstrated.

## 4 Conclusion

Synthetic biology relies on reusing standardised genetic DNA parts such as regulatory sequences, thus, having access to DNA parts that lead to genetic- and host-context independent expression is valuable. We show that the GeneEE approach can be utilised to generate regulatory sequences that function consistently in a variety of host- and genetic-contexts. In this study we cross-characterised ARESs in two different hosts using two fluorescent reporter genes. 3008 clones were screened in *V. natriegens* and 26 clones exhibiting a range of FIs were chosen for further use. We exchanged the initially used *sfGFP* reporter gene with *mCherry* and transformed *E. coli* and *V. natriegens* with the constructs. The resulting 52 constructs were used to drive expression of both genes in both *E. coli* and *V. natriegens*. By cross-characterising the clones, we identified ARESs that led to reliable expression in all host-reporter combinations, and ARESs that are functioning in a gene- or host-dependent manner. We also found one ARES which led to a significant increase in FI in a carbon source dependant fashion indicating that the reported method can be also used to create inducible ARESs. To conclude, we broaden the repertoire of known and described genetic elements available for synthetic biology research using *V. natriegens* as host microorganism.

## 5 Materials and Methods

### 5.1 Bacterial Strains and Growth Conditions

*E. coli* DH5*α* (Bethesda Research Laboratories) was used for cloning work and fluorescence measurements. *V. natriegens* strain ATCC 14 048 was gifted to you by the Blombach group at Technical University Munich, for which we are very grateful. *E. coli* carrying plasmids were cultivated in sterile LB medium (trypsin (10 g/L), yeast extract (5 g/L), NaCl (5 g/L)) supplemented with 25 mg/L chloramphenicol. *V. natriegens* carrying plasmids were cultivated in LBN medium (trypsin (10 g/L), yeast extract (5 g/L), NaCl (15 g/L)) supplemented with 12.5 mg/L chloramphenicol. Both strains were grown at 37°C, and for liquid cultures, flasks or tubes were shaken at 225 rpm. *V. natriegens* plates were stored at room temperature since the bacteria are sensitive to refrigeration (Weinstock et al., 2016). M9N minimal medium consisted of [per litre (Wu et al., 2020)]: 200 ml of sterile 1X M9N minimal medium (5x M9N medium: KH_2_PO_4_, 15 g/L, NaCl, 2.5 g/L, Na_2_HPO_4_, 33.9 g/L, NH_4_Cl, 5 g/L) supplemented with 2 mM MgSO_4_, 0.1 mM CaCl_2_, and 15 g/L NaCl. The carbon source was supplemented in a concentration of 20 g/L. The tested modified CGXII medium consisted of [per litre (Hoffart et al., 2017)]: 5 g (NH)_2_SO_4_, 15 g NaCl, 1 g KH_2_PO_4_, 1 g K_2_HPO_4_, 0.25 g MgSO_4_, 0.01 g CaCl_2_, 16.4 mg FeSO_4_⋅7H_2_O, 10 mg MnSO_4_⋅H_2_O, 0.3 mg CuSO_4_⋅5H_2_O, 1 mg ZnSO_4_⋅7H_2_O, and 0.02 mg NiCl_2_⋅6H_2_O, 10 g carbon source.

Glycerol stocks were prepared with a final concentration of 20% glycerol and were stored at -80°C. To prepare electrocompetent *V. natriegens*, a protocol provided by the Blombach group was followed. 5 ml of liquid over-night (ON) culture of *V. natriegens* were grown at 37°C, shaking at 225 rpm. The next day, the 5 ml ON culture is used to inoculate 250 ml main culture and grown to an OD_600_ = 0.5, which can be as quickly as 30 min later depending on density and viability of the ON culture. When target OD_600_ is reached, incubate cells on ice for 15 min. Centrifuge cells at 4500 rpm for 20 min at 4°C. Discard supernatant and resuspend cells in 10 ml electroporation buffer (680 mM sucrose, 7 mM K2HPO4; set pH to 7 with 32% HCl (few droplets)). Fill to 70 ml of electroporation buffer. Centrifuge cells at 4500 rpm for 15 min at 4°C. Repeat electroporation buffer wash step two more times. Finally, resuspend your cells so that you reach a final OD_600_ = 16. Divide the cell suspension into aliquots of 50 *μ*L and snap freeze in liquid nitrogen and store at -80°C. For electroporation, thaw aliquots on ice for 10 min. Add 100–400 ng of plasmid (max. 2 *μ*L) to the cells, mix and incubate on ice for 15 min. Transfer to a sterile and ice cold electroporation cuvette (2 mm gap width), keep on ice until electroporation. Pulse at 1,800 V, 25 *μ*F, 200 *Ω* and immediately resuspend cells in 5 ml of BHIN [Brain-Heart infusion (37 g/L), NaCL (15 g/L)]. It is important to use BHIN in this step and not LBN. To our experience amount of cfu drops drastically when LBN is used in the recovery step. Recover cells for 2 h at 37°C, shaking at 225 rpm. Depending on the amount of colonies wanted, take sample directly from the recovery medium and plate out or centrifuge cells and resuspend in smaller volume.

### 5.2 DNA Manipulations

Standard recombinant DNA procedures were performed to create the base plasmid pACYC184-sfGFP. The pACYC184 vector served as a backbone to insert a fragment containing the *Bacillus methanolicus* derived mdh regulatory region including the *mdh* promoter and 5′ UTR (Jakobsen et al., 2006; Irla et al., 2016), and DNA sequence coding for a superfold Green Fluorescent Protein (sfGFP). The resulting plasmid was then used to for further cloning. The *mdh* regulatory region was replaced with a regulatory region comprising Anderson promoter J23101^9^, ribozyme RiboJ (Lou et al., 2012), and a ribosome binding site B0046^10^. To create artificial promoter-5′ regulatory sequences, the regulatory region was removed and replaced with DNA stretches of fully randomised nucleotide sequence with or without SD-motif. The random nucleotide sequence was synthesised as single stranded DNA by IDT. The random nucleotide sequence DNA strands contained two defined flanking regions serving two purposes: The flanking regions were used as primer binding sites during PCR, which was run to amplify the random sequence strands, and to create double stranded DNA stretches for subsequent cloning. The flanking regions contain two unique BsaI sites (one on each end), allowing scar-less Golden Gate cloning. The synthetic DNA was amplified using a shortened PCR protocol with only 10 cycles. Using more than 10 PCR cycles resulted in a smear on the gel at larger sizes than expected. This suggests that the random DNA sequence fragments start binding each other later in the process, serving as primers to further elongation. This can be prevented by using 10 or less cycles in the amplification step. The PCR product was purified using the QIAGEN PCR purification kit. Next, the pACYC184-sfGFP plasmid backbone was amplified via PCR. For this, a primer pair was used that amplified the whole vector and only excluded the promoter-5′ region upstream of sfGFP. At the same time, the primers introduce defined flanking regions that contain unique BsaI sites that fit to the BsaI sites of the insert. The PCR fragment was digested with DpnI over night at 37°C to remove any parental plasmid and the digestion mix was purified. Subsequently, Golden-Gate cloning was performed using 75 ng of both insert and backbone and 30 cycles of alternating 5 min temperature steps of 37 and 16°C (Potapov et al., 2018). The Golden-Gate mix was used to transform *E. coli* DH5*α* (10 *μ*L mix per 100 *μ*L chemical competent cells) and plated on LB plates containing 25 mg/L chloramphenicol. Plates were incubated over night at 37°C and inspected the next day. Colonies were counted (ca. 10.000 per library). A colony PCR and subsequent Sanger sequencing of the ARES region was performed with 10 randomly selected clones to confirm correctness of constructs. All 10 clones showed the expected band size on an agarose gel after the colony PCR (data not shown). The sequencing results confirmed correct insertion of the insert of nine of the constructs. The *E. coli* clone libraries were scraped from the plates and plasmids were isolated using the QIAGEN Plasmid MiniPrep Kit. The plasmid library was electroporated into *V. natriegens* (protocol provided by Blombach group). The size of each of the libraries in *V. natriegens* was ca. 15,000 clones. To exchange sfGFP with mCherry, first the positive controls were established by exchanging sfGFP with mCherry. We used a shorter version of mCherry to prevent the background expression of the short-form mCherry that is encoded within the standard long version of mCherry Fages-Lartaud et al. (2021). First the vector was PCR-amplified, excluding sfGFP. mCherry was PCR-amplified from a different plasmid and cloned into the vector using Gibson cloning with a self-made Gibson mix (Gibson et al., 2009). The positive-control mCherry plasmid then was used to amplify the vector without the regulatory regions upstream of mCherry. In its stead we inserted the different ARESs from previously selected clones. The ARESs were PCR-amplified from their respective plasmids and inserted into the mCherry vector via Gibson cloning. Constructs were confirmed by Sanger sequencing performed by Eurofins. Negative control plasmids were created by PCR-amplifying the vector without the regulatory region upstream of sfGFP or *mCherry* and then phosphorylating the PCR product using T4 PNK (NEB): Use up to up to 75 pmol of 5′ termini of DpnI digested and purified PCR product, 1 *μ*L T4 ligase buffer (NEB), 0.25 *μ*L T4 PNK, fill with water to 10 *μ*L. Incubate at 37°C for 30 min, heat inactivate for 20 min at 65°C. The phosphorylated vector was blunt end ligated using T4 ligase (NEB). For this, take the 10 *μ*L of the phosphorylation step and add following ingredients: 2 *μ*L T4 ligase buffer (NEB), 1 *μ*L T4 ligase (NEB), fill to 20 *μ*L with water. Incubate for 2 h at room temperature, heat inactivate for 10 min at 65°C, then use for transformation (10 *μ*L per 100 *μ*L chemical competent *E. coli*). Constructs were confirmed by Sanger sequencing performed by Eurofins. All sequences of ARES are in Supplementary Table 1. Plasmid maps are available in GeneBank format in the Supplementary Material. To create any of the ARES plasmid maps, replace “200N” in the “pACYC-sfGFP 200N” file with the ARES found in Supplementary Table 1.

### 5.3 Selection and Characterisation of ARES Strength

Clones were selected by measuring fluorescence of ca. 3000 randomly selected clones in liquid culture. For this, *V. natriegens* carrying library plasmids were randomly selected and grown in a 96-well plate containing LB and chloramphenicol. The 96-well plate was incubated at 37°C and 850 rpm and sfGFP florescence was measured (485–520 nm). The results were used to assess hit-rate and select 20 unique clones in a wide range of fluorescence levels. The strength of selected ARESs was assessed using sfGFP and mCherry fluorescence intensity. For this, over night cultures of the selected clones were grown in 96-well format over night. A replicator was used the next day to stamp from the over-night culture into a 1 ml-well 96-well format. Cells were grown at 37°C and 850 rpm. Growth and fluorescence was measured every hour for *V. natriegens* and every 2 h for *E. coli* until growth reached stationary phase. Fluorescence and OD_600_ were measured using a TECAN SpectraMax reader. For sfGFP, the excitation wavelength was 485 nm, and emission wavelength was 520 nm. For mCherry excitation wavelength was 585 nm, and emission wavelength was 620 nm. Fluorescence intensity at the end of exponential grwoth phase was used for the cross-characterisation.

### 5.4 Testing and Selection of ARESs Leading to Inducible Fluorescence

Inducible fluorescence screening was performed on minimal media agar plates containing different carbon sources. The selected carbon sources were d-glucose, sucrose, fructose, maltose, L-arabinose, glycerol, glucosamine hydrochloride and succinic acid. For this, the *V. natriegens* 200 + SD and 50N + SD libraries were plated out onto minimal media agar plates. When growing *V. natriegens* on rich media plates, it is difficult to identify colonies that emit green fluorescence. However, when grown on minimal media plates, fluorescent colonies become visible under UV light after growing for more than 3 days. Thus, selection of fluorescent colonies growing on minimal media plates was conducted by growing the *V. natriegens* libraries on the different minimal media plates containing the distinct carbon sources and checking the plates daily under a UV light. Green fluorescent colonies were selected, plasmids extracted and ARESs sequenced using Sanger sequencing. Six unique clones were identified, which were added to the pool of previously selected 20 clones from rich medium. The six clones were cross-characterised with the other 20 clones, but were also subjected to an inducible fluorescence experiment. The *V. natriegens* clones were cultivated in eight different M9N minimal media and characterised with sfGFP and in *V. natriegens*. The different M9N media contained distinct carbon sources: d-glucose, sucrose, fructose, maltose, L-arabinose, glycerol, glucosamine hydrochloride and succinic acid.

### 5.5 Fluorescence Microscopy

Fluorescence microscopy was conducted using a Zeiss Axio Imager.Z2 with a ×20 magnification lense. A GFP channel (excitation at 488 nm and emmission at 509 nm) was used to visualise fluorescence. The phase contrast channel was used to make cells visible. Images were taken with an ApoTome camera (Zeiss) camera and processed with the ZEN software. *V. natriegens* cells moved substantially during the picture taking process, resulting in incorrect overlays. Heat fixation was tried and showed that this procedure destroyed integrity of sfGFPs. Instead, a combination of low volume (<2 *μ*L) and mechanical pressure onto the cover slip were successful in immobilising the cells.

### 5.6 Analysis of Sigma Factors and ARESs

Genomic sequences were derived from NCBI GenBank. Genome of *V. natriegens* (accession ID: CP016345 and NZ_CP016346.1) and *E. coli* (accession ID: U00096.3). To align protein sequences of sigma factors, EMBOSS Stretcher was used. BPROM, CNNPromoter_b and Salis Promoter calculator were used to predict transcription start sites. The results from CNNPromoter_b were used for downstream analysis. iPromoter-2L was used to analyse the prompter sequences determined by CNNPromoter_b. 81 nucleotides upstream of the transcription start site were used to run iPromoter-2L. To create weighted sequence alignment, weblogo was used. For the use of weblogo, sequences needed to be the same length. Thus, sequences were trimmed to the same length, using the shortest sequence as target length.

### 5.7 Statistical Analysis

For the statistical analysis of change of FI in the minimal media experiment, FI values were used to conduct pairwise one-tailed T-tests. FI of pACYC-sfGFP ARES 4 clone cultivated in the different minimal media were compared to FI of pACYC-sfGFP ARES 4 cultivated in rich medium. The amount of asterisks in the figure indicates the significance of the result of the one-tailed T-tests: *p-value < 0.05; **p-value < 0.01; ***p-value < 0.001; ****p-value < 0.000 1. The same statistical analysis was performed to compare FI of clones carrying pACYC-sfGFP ARES 4 cultivated in different media to FI of clones carrying the positive control plasmid pACYC-mdh-sfGFP cultivated in the same medium. For the statistical analysis of correlation of measured FI values with predicted transcription and translation rates, Spearman’s rank correlation was performed. The Spearman’s rank correlation can be used to assess the statistical dependence between raked variables. Thus, first, the data was ranked in ascending order. Then, the ranked values were correlated. For this, the ranked values of measured FIs were correlated with ranked values of the predicted transcription rates or the predicted translation rates. The resulting values are Spearman’s rank correlation coefficients.

## Data Availability

The original contributions presented in the study are included in the article/Supplementary Material, further inquiries can be directed to the corresponding author.

## Footnotes

1
http://parts.igem.org/Promoters/Catalog/Anderson.

2
http://parts.igem.org/Part:BBa_B0064.

3
https://www.ebi.ac.uk/Tools/psa/emboss_stretcher/.

4
https://blast.ncbi.nlm.nih.gov/Blast.cgi.

5
http://www.softberry.com/berry.phtml?topic=bprom&group=programs&subgroup=gfindb.

6
http://www.softberry.com/berry.phtml?topic=cnnpromoter_b&group=programs&subgroup=deeplearn.

7
https://weblogo.berkeley.edu/logo.cgi.

8
https://www.usclimatedata.com/climate/sapelo-island/georgia/united-states/usga0501.

9
http://parts.igem.org/Promoters/Catalog/Anderson.

10
http://parts.igem.org/Part:BBa_B0064.
