# Supplementary material for: Identification and Cross-Characterisation of Artificial Promoters and 5′ Untranslated Regions in Vibrio natriegens
Source: Front Bioeng Biotechnol. 2022 Jan 27;10:826142. doi: 10.3389/fbioe.2022.826142 (PMC8830501; doi:10.3389/fbioe.2022.826142)
Supplement: Supplementary file 1 [file DataSheet2.PDF]

```
EcU00096.3_rrsH_b0201              CTGCGGTTGGATCACCTCCTTA-------
EcU00096.3_rrsA_b3851              CTGCGGTTGGATCACCTCCTTA-------
EcU00096.3_rrsB_b3968              CTGCGGTTGGATCACCTCCTTA-------
EcU00096.3_rrsE_b4007              CTGCGGTTGGATCACCTCCTTA-------
EcU00096.3_rrsG_b2591              CTGCGGTTGGATCACCTCCTTA-------
EcU00096.3_rrsC_b3756              CTGCGGTTGGATCACCTCCTTA-------
EcU00096.3_rrsD_b3278              CTGCGGTTGGATCACCTCCTTA-------
VnCP016345.1_BA890_13075           CTGGCGCTGGATCACCTCCTTATACGATG
VnCP016345.1_BA890_02615           CTGGCGCTGGATCACCTCCTTATACGATG
VnNZ_CP016346.1_BA890_RS20575      CTGGCGCTGGATCACCTCCTTA-------
VnCP016345.1_BA890_13940           CTGGCGCTGGATCACCTCCTTATACGATG
VnCP016345.1_BA890_12615           CTGGCGCTGGATCACCTCCTTATACGATG
VnCP016345.1_BA890_14445           CTGGCGCTGGATCACCTCCTTATACGATG
VnCP016345.1_BA890_00105           CTGGCGCTGGATCACCTCCTTATACGATG
VnCP016345.1_BA890_13060           CTGGCGCTGGATCACCTCCTTATACGATG
VnCP016345.1_BA890_13925           CTGGCGCTGGATCACCTCCTTATACGATG
VnCP016345.1_BA890_14135           CTGGCGCTGGATCACCTCCTTATACGATG
VnCP016345.1_BA890_14155           CTGGCGCTGGATCACCTCCTTATACGATG
VnCP016345.1_BA890_14650           CTGGCGCTGGATCACCTCCTTATACGATG
```

Supplementary Figure 1: Sequence alignment of 3´ of the 16S rRNA sequences of *V. natriegens* (Vn) and *E. coli* (Ec). After Ec/Vn indicators follow the accession numbers (U00096.3, CP016345.1, and CP016346.1), followed by the gene indicator (“rrsH_b0201”, “BA890_13940”, etc.). The conserved anti-SD-sequence is highlighted in yellow.

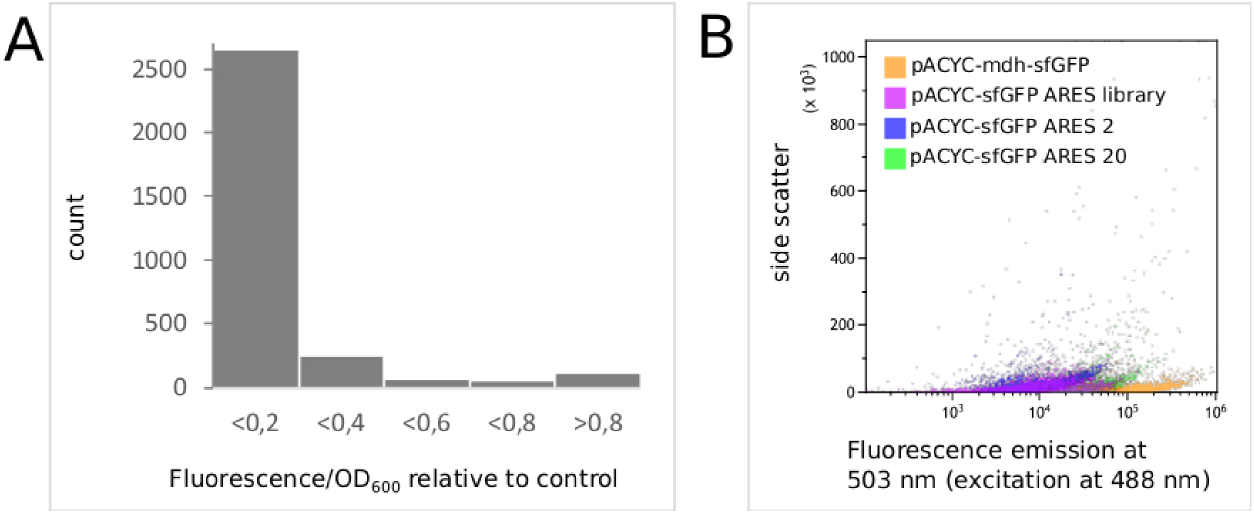

Supplementary Figure 2: Overview of library clone distribution. (**A**) Plot showing the distribution of measured sfGFP fluorescence intensities of the screened 3000 *V. natriegens* clones. Of the 3000 clones, 2500 clones carry 200N+SD library plasmids and 500 clones carry 50N+SD library plasmids. (**B**) Plot showing results of a flow cytometer experiment with different select *V. natriegens* clones. Each dot represents an event (a cell). The y-axis shows cells size, the x-axis shows fluorescence emission on a logarithmic scale. The different colors represent: Positive control pACYC-mdh-sfGFP (orange), 200N+SD library (pink), pACYC-sfGFP ARES 2 (blue), pACYC-sfGFP ARES 20 (green).

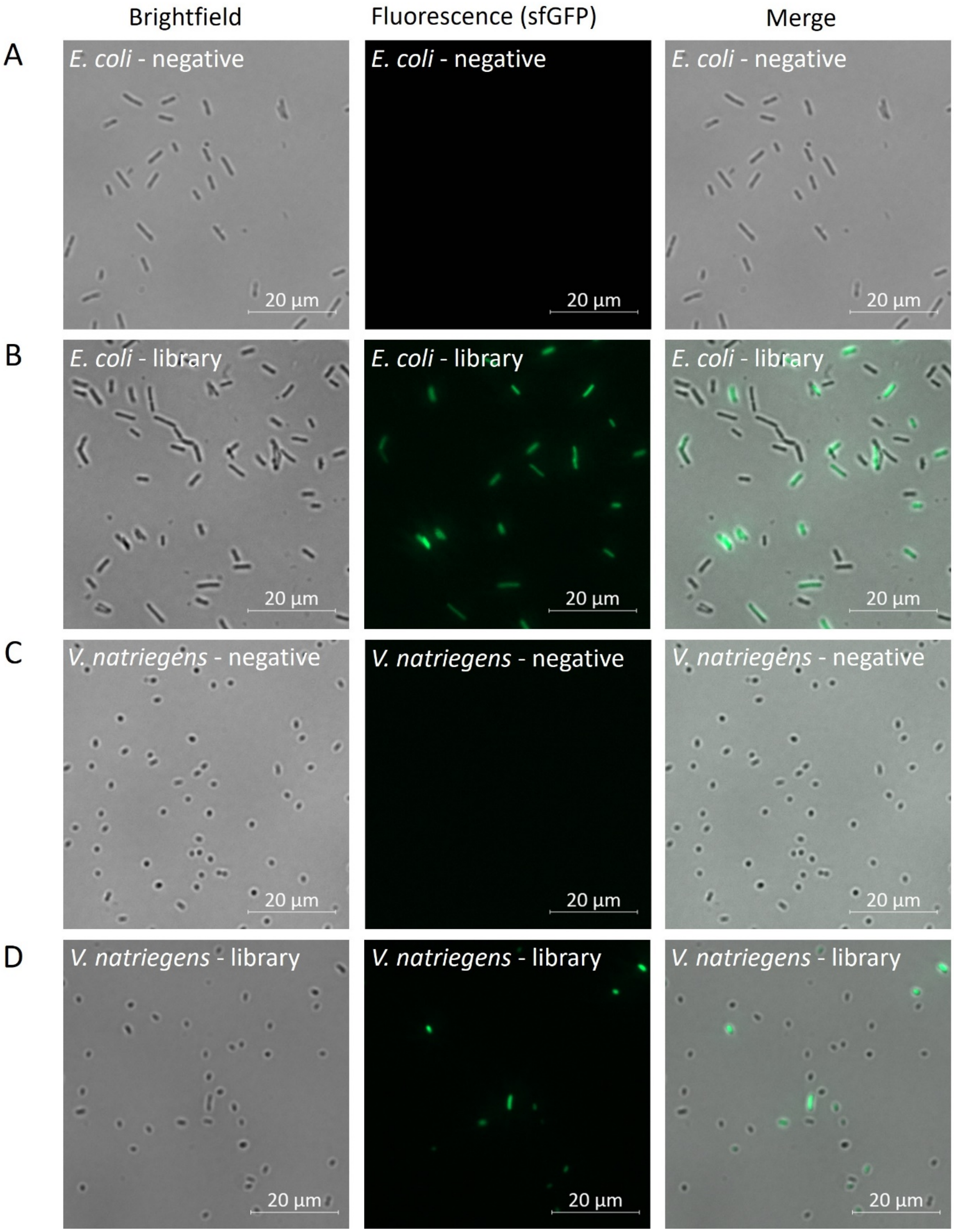

Supplementary Figure 3: Microscopy images taken of *E. coli* and *V. natriegens*. The images were taken with a Brightfield channel (left), fluorescence channel (middle) and then both the images were overlayed (right). Images were taken of clones carrying the negative control plasmid pACYC-sfGFP-negative control in *E. coli* (**A**) or *V. natriegens* (**C**) or carrying the plasmid library 200N+SD in *E. coli* (**B**), or *V. natriegens* (**D**).

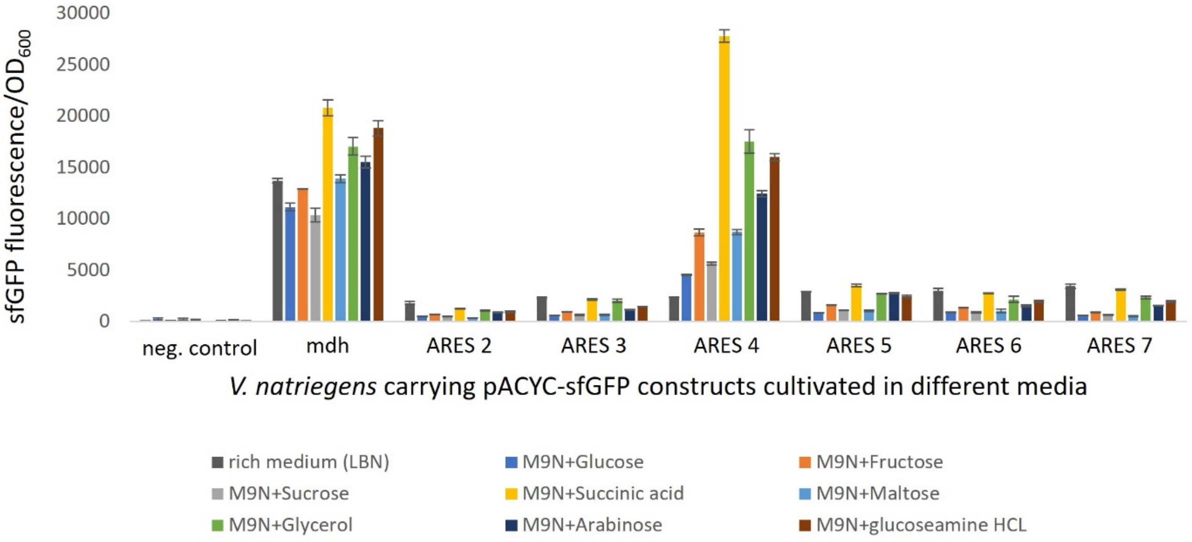

Supplementary Figure 4: Comparison of sfGFP FI of *V. natriegens* cultivated in M9N supplemented with different carbon sources as indicated by colour. The negative control exhibits negligible fluorescence intensity. FI changes in a medium-dependent fashion. Error bars indicate the standard deviations calculated from three biological replicates.

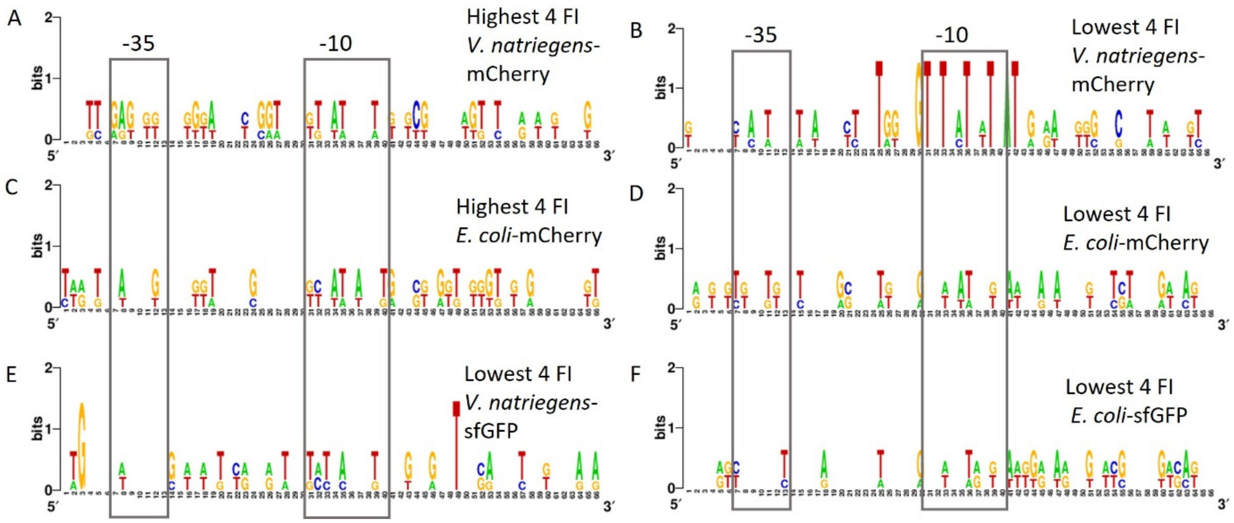

Supplementary Figure 5: Sequence logo created from trimmed aligned predicted promoter sequences. The sequence logo represents nucleotide biases in specific positions by size. The taller the nucleotide, the more frequently the nucleotide appeared in that position of the input sequences. Different alignments were made: (**A**) promoter sequences of the four *V. natriegens* clones with highest mCherry FI (clones 13, 15, 18, 21), (**B**) promoter sequences of the four *V. natriegens* clones with lowest mCherry FI (clones 3, 5, 6, 7), (**C**) promoter sequences of the four *E. coli* clones with highest mCherry FI (clones 16, 23, 25, 26). (**D**) promoter sequences of the four *E. coli* clones with lowest mCherry FI (clones 5, 8, 17, 20). (**E**) promoter sequences of the four *V. natriegens* clones with lowest sfGFP FI (clones 1, 2, 3, 4). (**F**) promoter sequences of the four *E. coli* clones with lowest sfGFP FI (clones 2, 5, 17, 20). The -10 and -35 regions are indicated.

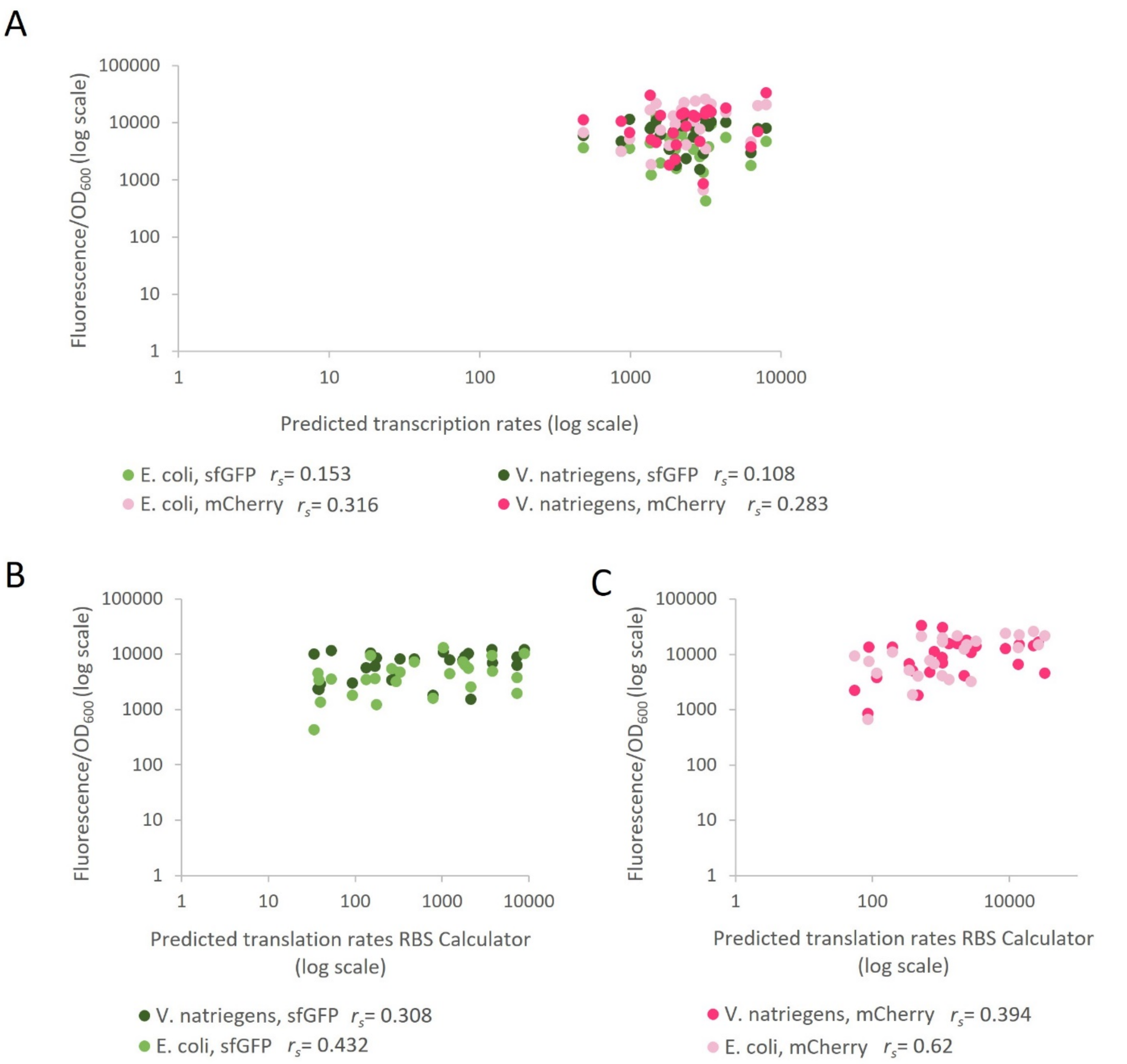

Supplementary Figure 6: Plots of correlated predicted transcription and translation rates from Salis promoter calculator and RBS calculator with measured fluorescence intensities (fluorescence/OD_{600}; FI). (**A**) plot of correlation of predicted transcription rates with FI of the different clones. (**B**) Plot of correlation of predicted translation rates with FI of clones producing sfGFP. (**C**) plot of correlation of predicted translation rates with FI of clones producing mCherry. Spearman's rank correlation coefficients (r_s) are indicated next to the host-reporter names.
